# Effect of the Latent Reservoir on the Evolution of HIV at the Within- and Between-Host Levels

**DOI:** 10.1371/journal.pcbi.1005228

**Published:** 2017-01-19

**Authors:** Hilje M. Doekes, Christophe Fraser, Katrina A. Lythgoe

**Affiliations:** 1 Department of Infectious Disease Epidemiology, Imperial College London, London, United Kingdom; 2 Theoretical Biology, Utrecht University, Utrecht, The Netherlands; 3 Oxford Big Data Institute, Li Ka Shing Centre for Health Information and Discovery, Nuffield Department of Medicine, University of Oxford, Oxford, United Kingdom; 4 Department of Zoology, University of Oxford, Oxford, United Kingdom; ETH Zurich, SWITZERLAND

## Abstract

The existence of long-lived reservoirs of latently infected CD4+ T cells is the major barrier to curing HIV, and has been extensively studied in this light. However, the effect of these reservoirs on the evolutionary dynamics of the virus has received little attention. Here, we present a within-host quasispecies model that incorporates a long-lived reservoir, which we then nest into an epidemiological model of HIV dynamics. For biologically plausible parameter values, we find that the presence of a latent reservoir can severely delay evolutionary dynamics within a single host, with longer delays associated with larger relative reservoir sizes and/or homeostatic proliferation of cells within the reservoir. These delays can fundamentally change the dynamics of the virus at the epidemiological scale. In particular, the delay in within-host evolutionary dynamics can be sufficient for the virus to evolve intermediate viral loads consistent with maximising transmission, as is observed, and not the very high viral loads that previous models have predicted, an effect that can be further enhanced if viruses similar to those that initiate infection are preferentially transmitted. These results depend strongly on within-host characteristics such as the relative reservoir size, with the evolution of intermediate viral loads observed only when the within-host dynamics are sufficiently delayed. In conclusion, we argue that the latent reservoir has important, and hitherto under-appreciated, roles in both within- and between-host viral evolution.

## Author Summary

During HIV infection, a small proportion of infected cells containing integrated proviral DNA enter a long-lived resting phase, thus creating latent reservoirs of virus. These reservoirs are a major barrier to curing HIV and have been studied intensively in this light. Latent reservoirs might also affect viral dynamics during untreated infection, because they provide an archive of old viral variants that can re-enter circulation at a later time upon activation of latently infected cells. We developed a mathematical model to investigate how reservoir dynamics affect the evolution of the virus within single hosts, and find that the presence of a latent reservoir can severely delay within-host evolutionary dynamics. This delay increases with the relative size of the reservoir and the rate at which latently infected cells proliferate. By nesting this within-host model into an epidemiological model of host population dynamics, we show that the presence of latent reservoirs can also influence the population-level evolution of the virus. In contrast to predictions made by previous models we find that population-level adaptation to maximise transmission between hosts, rather than within-host fitness, can occur, but only if adaptation of the virus within hosts is sufficiently slowed down by the presence of latent reservoirs.

## Introduction

Shortly after the introduction of anti-retroviral therapy (ART) to treat HIV infected individuals, viral reservoirs, in the form of long-lived CD4+ T cells containing integrated proviral DNA, were identified in infected patients [1,2]. These reservoirs are established soon after infection [3,4], are long-lived [5], and virus can re-emerge from the reservoirs after months or even years of latency [6]. As such, latent reservoirs represent the major barrier to curing HIV [7,8]. Although reservoirs of latent viruses have been extensively studied in the light of treatment and viral eradication, including several modelling studies (see [9] and [10] for recent reviews), we have little understanding of how latent reservoirs influence viral dynamics during untreated infection, and in turn how this might affect viral evolution at the epidemiological scale.

Viral lineages in the reservoir are expected to be evolutionarily very stable because proviruses only replicate when the host cell divides, and since this uses the host-cell replication machinery, mutation rates are extremely low. The reservoir is therefore likely to provide an archive of “ancestral” viral strains that were circulating earlier during the infection. If these archived viruses are reactivated months, or even years, after infection, their presence might have a profound influence on within- and between-host dynamics. Using a population-genetic modelling approach Kelly *et al*. showed that cycling through the reservoir can lower the within-host neutral rate of HIV evolution [11,12]. In a model of drug resistance, Ward *et al*. furthermore showed that a latent reservoir can provide memory of non-resistant viral strains for up to several years after treatment is started [13], and recently, Immonen *et al*. showed that substitution rates among within-host HIV lineages are overdispersed, which is consistent with the circulating of virus through a reservoir [14]. Using a simulation model of within-host dynamics, it has also been predicted that due to recombination a significant fraction of circulating viral strains will have fragments of their genome that have been latent for some time during infection [15]. Moreover, there has been speculation that the transmission of ancestral strains could explain the lower rates of HIV evolution observed at the epidemiological scale compared to within individuals [16–19], and the unexpectedly high heritability of HIV set-point viral load despite considerable within-host evolution between transmission events [20,21].

Here, we present a quasispecies model of within-host HIV evolutionary dynamics that incorporates a latently infected reservoir. Of note, we find that the presence of a latent reservoir can severely delay the within-host evolution of the virus. The extent of this delay is primarily determined by the replication rates of competing strains, the activation rate of latently infected cells, the relative size of the reservoir compared to the number of active infected cells (which we estimate from published data [1,22–26] to be between 0.06 and 3.1), and the extent to which cells within the reservoir proliferate. Since it is reasonable to speculate that all of these factors vary considerably among infected individuals, the influence of the reservoir in different individuals is likely to be very heterogeneous, and, if recombination is pervasive and selection pressures differ across the HIV genome, might also vary among sites [15].

Next, we nest the within-host model into a standard model of HIV epidemiological dynamics, enabling us to determine the effects of within-host evolution, and importantly the reservoir, on between-host evolutionary dynamics. If viral strains differ in their within-host fitness, variants with a higher *in vivo* replication rate will be favoured within a given host. Indeed, the replicative capacity of viral populations (as measured by *in vitro* competition assays) has been found to increase over the course of an HIV infection, albeit slowly [27,28]. It was also shown that individuals infected with a virus with a high replicative capacity tend to have a high viral load and vice versa [28,29], with the replicative capacity either predicted from the Pol sequence of the circulating virus [28] or measured by an *in vitro* competition assay [29]. Furthermore, in a study of 149 transmission pairs a weak positive correlation was found between the replicative capacity conferred by the Gag sequence of the virus that initiated an infection and the set-point viral load (spVL) [30], the relatively stable concentration of viral particles in the blood during the chronic phase of HIV infection. The spVL, in turn, is a predictor of the severity of infection, or virulence, since individuals with a high set-point tend to progress to AIDS faster [31,32].

As well as being a predictor for the duration of infection, set-point viral load is also correlated with the transmission rate [33,34]. Hence, infected individuals with a low set-point viral load (who, because of the correlations described above, are likely infected with a strain that has a low replicative capacity) will live long but will infect few other individuals because their transmission rate is low, while individuals with a high viral load will have a high transmission rate during the infection but will also cause few new infections because progression to AIDS is fast. Under this trade-off between duration of the infection and the transmission rate, it has been calculated that the transmission potential (defined as the number of new infections caused by an infected individual over the entire infectious period) is maximised when set-point viral loads are intermediate, i.e. log(spVL) ≈ 4.5 [35,36]. The distribution of set-point viral loads found in populations of infected individuals is centered around these intermediate values, suggesting that HIV virulence might have evolved to maximise between-host transmission [35–37]. This is surprising, since HIV evolves rapidly within hosts and durations of infections are long, and so the virus should be expected to evolve to maximise within-host fitness (e.g. replication rate) at the expense of between-host fitness, an outcome known as ‘short-sighted’ evolution [38]. Previous mathematical and computational studies have explored this interplay between within- and between-host selection, either by including within-host selection for high replication rate, which was in turn directly coupled to higher virulence as described above [20], or by including within-host selection for immune escape mutations in a heterogeneous host population and hence allowing the virus to adapt to each new host [39]. In both cases, for realistic within-host selection pressures and mutation rates, the within-host selection process was found to dominate between-host selection, and hence between-host level adaptation was not predicted to occur [20,39].

Here, we find that delays in within-host dynamics caused by the presence of a reservoir can have a large impact on how the virus evolves at the epidemiological level, and can, under certain parameter conditions, be substantial enough to explain evolution towards intermediate levels of virulence that maximise transmission. We therefore contend that it is important to consider reservoir dynamics not only in patients on antiretroviral therapy, but also in untreated individuals with ongoing active viral replication. Understanding how the virus evolves under different levels of selection is crucial if we want to better describe the course of epidemics, and predict the evolutionary impacts of potential intervention measures.

## Results

First, we investigate the effect of the latent reservoir on the within-host evolution of HIV by studying a model of within-host evolutionary dynamics, before nesting the within-host model into an epidemiological framework.

### Within-host dynamics

#### Model

**Fig 1:**
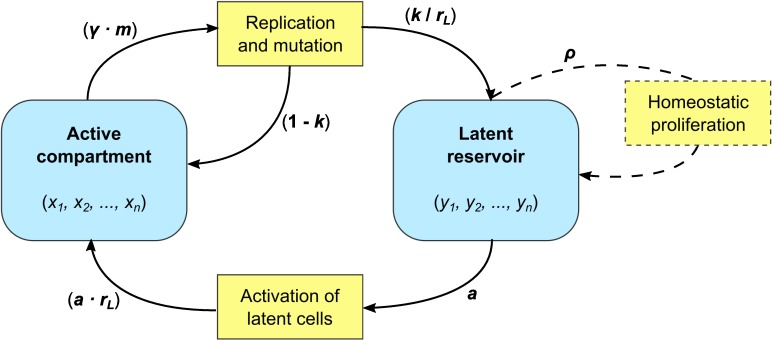
Schematic overview of the within-host model. The frequency of *n* different strains in the active compartment and latent reservoir (*x*_*j*_ and *y*_*j*_ respectively; *j =* 1, …, *n*) are tracked. In the active compartment, strain-*j* virus replicates with strain-specific replication rate γ_*j*_ and can mutate into other strains *i* with probability *m*_*ij*_. A small fraction, *k*, of newly infected cells become resting cells and move into the latent reservoir. Latent cells are reactivated at rate *a* per day. The effect of cells moving between the two compartments on the strain frequencies in these compartments depends on the relative size of the reservoir compared to the active compartment, *r*_*L*_. The relative reservoir size is assumed to be constant throughout infection, maintained by a balance between inflow and outflow of cells, or by proliferation of latently infected cells if the outflow rate from the reservoir exceeds the entry rate. Specifically, the homeostatic proliferation rate is given by $\rho =a-\frac{k}{r_{L}}$ (see Methods for derivation). See *Table 1*for model parameter values.

We developed a mathematical model that describes the cycling of viral strains through an active compartment and a latent reservoir (*Fig 1*). The active compartment represents infected actively replicating CD4+ T cells, while the reservoir represents long-lived latently infected CD4+ T cells. Extending the model proposed by Lythgoe *et al*. [20], we use two coupled quasi-species equations [40] to describe the frequency of the different viral strains in these compartments. We chose to consider viral frequencies rather than the absolute number of infected cells since tractable models that produce realistic profiles of infection and can also accommodate the distribution of viral loads found among infected individuals do not currently exist [41]. A full description and derivation of the model are given in the Methods. In short, viral replication takes place in the active compartment at a strain-dependent replication rate, and with mutation occurring during replication. Most of the newly infected cells are assumed to directly feed back into the active compartment. However, upon infection a small fraction, *k*, of these cells enter a long-lived resting phase and become part of the reservoir. Cells in the reservoir are reactivated at rate *a* per day, at which point they re-enter the active compartment.

Since we describe the frequency of viral strains, rather than the absolute number of infected cells, we have to account for a potential size difference between the two compartments. For example, if there are more actively infected than latently infected cells, then a single cell carrying a specific strain that moves from the reservoir to the active compartment will have a smaller effect on the viral strain frequency distribution in the active compartment than it had on the frequency distribution in the reservoir. We therefore define the relative reservoir size *r*_*L*_ to be the ratio between the number of latently infected cells (absolute size of the reservoir) and the number of actively infected cells (absolute size of the active compartment), and use this parameter to translate changes in the frequency distribution in one compartment to the appropriate changes in the other compartment (see *Fig 1*). *In vivo* measurements indicate that the reservoir is filled up quickly after infection, both in human patients [3] and in rhesus monkeys [4]. Because viral load remains relatively constant during the chronic phase of an HIV infection, we assume that the size of the active compartment and the reservoir, and therefore also the relative reservoir size, are constant during chronic infection. Since with existing data it is only possible to estimate the relative reservoir size during chronic infection (see below), we furthermore make the simplifying assumption that the relative reservoir size in acute and late infection is the same as during chronic infection. We will however show that relaxing this assumption has almost no effect on the results.

In the absence of proliferation of cells in the reservoir, the assumption of a constant reservoir size is met if the inflow of new latently infected cells equals the outflow by reactivation. If, however, parameters are chosen such that the outflow exceeds the inflow, we make the implicit assumption that the proliferation of cells within the reservoir maintains its size. The homeostatic proliferation rate is then given by *ρ* = outflow rate–inflow rate = $a-\frac{k}{r_{L}}$ (see Methods for derivation). Note that we neglect mutation during homeostatic proliferation, since the integrated latent HIV DNA will be replicated by the host cell machinery, which has high copying fidelity.

A full overview of the model parameters and their values is shown in *Table 1*. Within the active compartment, viral replication rates are assumed to be close to 1 replication cycle per day [42]. To model mutation and selection, we consider two scenarios: (i) a within-host selection model where the fastest replicating strain (strain *n*) has a 5% fitness advantage over the least fit strain (strain 1), reflected in an increased replication rate, and (ii) a within-host neutral model where all strains are of equal within-host fitness (but not necessarily of equal between-host fitness). For the first scenario we follow Lythgoe *et al*. [20] and use a fitness landscape with a single peak in which strain *j* can only mutate into strain *(j-1)* or *(j+1)*. Since this selection and mutation regime will lead to a monotonically increasing fitness trajectory, we call this a “hill-climb” fitness landscape. These mutations happen with probability 5 x 10^−5^ at replication [43,44]. For the second scenario, we let “strain” *j* represent a bin of all viral strains that have acquired *(j-1)* neutral mutations compared to the ancestral founder strain. Strain *n* then represents all viral strains carrying *≥n* mutations and is absorbing, meaning no mutations take place from strain *n* to strain *(n-1*). We consider a 300bp region of the genome (a typical read length in next generation sequencing studies) in which 1/3 of all mutations are assumed to be both synonymous and neutral, and hence the probability of acquiring a neutral mutation is 5 x 10^−3^, while the probability of reversion is again 5 x 10^−5^ [43,44]. Because we consider at most 16 strains (i.e. acquisition of at most 15 neutral mutations) we ignore saturation effects. Finally, we vary the probability of newly infected cells becoming latent, *k*, and the activation rate of latently infected cells, *a*. The probability of latency, *k*, is typically chosen to be 0.005 or less, and is never larger than 0.02, meaning that the vast majority of new cell infections lead to actively infected cells. The activation rate *a* is never bigger than 0.01 per day, thus ensuring that latently infected cells have a relatively long life span. As a consequence, homeostatic proliferation only occurs in our model at a low rate of less than one division per cell per 100 days (*ρ* < 0.01), if at all.

**Table 1:** Model parameters.

Parameter	Definition	Value
*γ*_*j*_	Replication rate of viral strain *j* (per day)	variable, [1.00–1.05]
*m*_*ij*_	Probability that strain *j* mutates into strain *i* during replication	5 x 10^−5^ iff |*i-j*| = 1 for the selection model;
		5 x 10^−3^ iff *i = j+1* and 5 x 10^−5^ iff *i = j-1* for the within-host neutral model.
*k*	Probability that a newly infected cell becomes latent	Variable, [0–0.02]
*a*	Activation rate of latent cells (per day)	Variable, [0–0.01]
*r*_*L*_	Relative size of the latent reservoir	Variable, [0.01–2]
*ρ*	Homeostatic proliferation rate of latent cells (per day)	$a-\frac{k}{r_{L}}$
*T*_*j*_	Duration of a type- *j* infection in the absence of natural mortality (years)	Variable, [1.9–21.4]
*α*_*j*_*(t)*	Time-dependent infectivity profile of individuals originally infected with strain *j*	Variable
*B*	Rate at which new susceptible individuals enter the host population (individuals per year)	200
*ν*	Natural death rate of hosts independent of infection status (per year)	0.02

**Fig 2:**
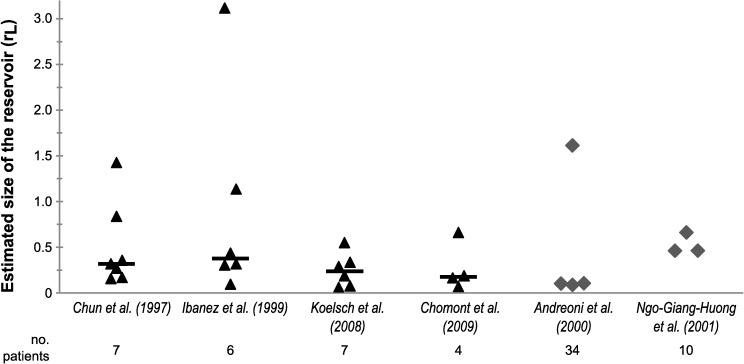
Estimated relative reservoir size *r*_*L*_ during chronic infection. Estimates were made using either a direct method based on the number of active and latent infected CD4+ T cells (data from Chun *et al*. [1]), or an indirect method using HIV DNA levels pre and post-treatment (data from [22–26]). For the left four studies, triangles represent single patients and the bar indicates the median. Two studies did not report data on individual patients, but on patient groups only. In these cases, the group mean [23] or median [24] was used to estimate *r*_*L*_ for each group.

Estimation of the relative reservoir size *r*_*L*_ poses a particular challenge, since studies reporting both the size of the latent reservoir and the number of actively infected cells are scarce, especially in non-treated patients. To our knowledge, only Chun *et al*. [1] report these kind of data, and only for the chronic, asymptomatic phase of infection. From their results we directly estimated the relative size of the reservoir during chronic infection for the 7 patients included in their study. In addition, we developed an indirect method to estimate the relative reservoir size using studies that report HIV DNA levels before and during antiretroviral treatment [22–26]. Using our direct and indirect methods, we arrive at estimates for the relative reservoir size that vary between 0.06 and 3.1 (*Fig 2,* see also Methods and *S1 Table* for full data per patient per study). In other words, our estimates vary between a setting in which the number of latently infected cells is approximately 20 times less than the number of actively infected cells and a setting in which there are 3 times as many latently infected cells as actively infected cells.

#### The presence of a reservoir can severely delay within-host evolutionary dynamics

Using the modified quasispecies model, we determined the influence of the reservoir on within-host evolutionary dynamics. HIV infections are generally established by a single viral genotype [45–47], and therefore the model was initialised with a single founder strain, with a further 15 strains generated through mutation giving a maximum of 16 strains. Importantly, we assume that the within-host dynamics are completely determined by the initial conditions, thus ignoring potential superinfections. We study the model both for the within-host selection case and the scenario in which within-host evolution is neutral.

**Fig 3:**
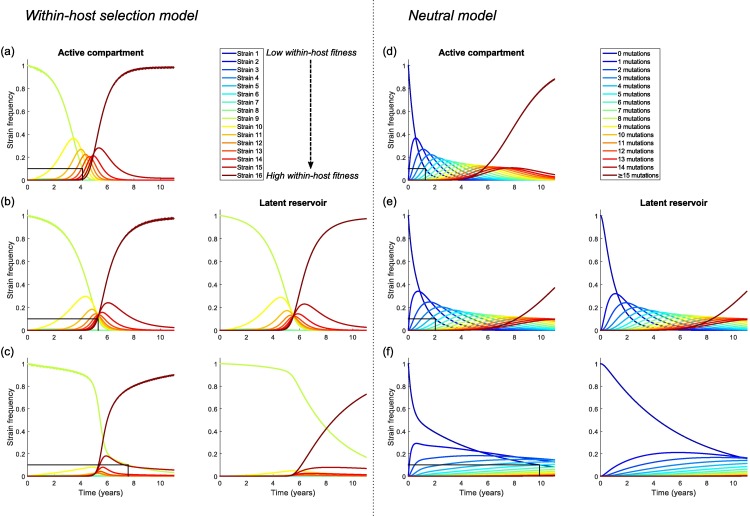
**Within-host dynamics for the within-host selection model (panels a-c) and the within-host neutral model (panels d-f)**, (a,d) in the absence of a reservoir (*k = a =* 0), (b,e) in the presence of a reservoir, but without homeostatic proliferation in the reservoir (*r*_*L*_ = 0.5, *k* = 5 x 10^−3^, *a* = 0.01 per day, *ρ* = 0 per day), and (c,f) in the presence of a reservoir, with a low level of homeostatic proliferation (*r*_*L*_ = 0.5, *k* = 5 x 10^−4^, *a* = 0.01 per day, *ρ* = 9 x 10^−3^ per day). The black line indicates the time at which the frequency of the initial strain has declined to 10%. The presence of a latent reservoir delays the within-host dynamics, and this delay becomes even more profound if there is a low level of homeostatic proliferation in the reservoir. The number of strains is *n* = 16. In the within-host selection model, strains have linearly increasing replication rates between *γ*_*1*_ = 1.0 and *γ*_*16*_ = 1.05 and the infection is initiated with strain 9. In the within-host neutral model, all strains have equal within-host fitness and strains are characterised by the number of neutral mutations they carry compared to the founder strain. In this case the last strain (carrying ≥15 mutations) is absorbing, i.e. there are no mutations out of this bin. All other parameter values are as stated in *Table 1*.

A comparison of model dynamics in the absence and presence of a reservoir is given in *Fig 3*, where *r*_*L*_ = 0.5 and *a* = 0.01 per day when the reservoir is present. First, consider the within-host selection scenario. We initiated the infection with strain 9, which has intermediate within-host fitness. In the absence of a reservoir (*Fig 3A*), as expected, strains with higher replication rates quickly increase in frequency in the viral population, until the population is dominated by the strain with the highest fitness. A similar process takes place if a latent reservoir is included in the model, where parameters are chosen such that there is no homeostatic proliferation of latently infected cells (i.e. the inflow and outflow from the reservoir are equal) (*Fig 3B*). However, when comparing the timescales we see that in the presence of a reservoir the founder strain persists at high frequency for longer and it takes more time for the system to approach equilibrium. Using the time it takes for the founder strain to drop to 10% frequency as our benchmark, the dynamics are delayed by approximately 1.5 years when the reservoir is included in the model. This large effect can be understood by recognising that although viral lineages only rarely enter and exit the reservoir, once in the reservoir they remain there for a long period of time (with a mean generation time in the reservoir of *1/a* days). Assuming a viral generation time of 1 day in the active compartment, and no homeostatic proliferation, the overall mean generation time will approach *(1-k) + k/a* days as infection progresses [11]. Note that because of our assumption of no homeostatic proliferation in the reservoir, we set the inflow (*k/r*_*L*_) equal to the outflow (*a*), i.e. we assume that the relative reservoir size *r*_*L*_ = *k/a*. Hence, in the absence of homeostatic proliferation the mean generation time will be approximately equal to 1 + *r*_*L*_ (for *k* << 1), and therefore we should expect the cycling of virus through the latent reservoir to drastically delay the within-host evolutionary dynamics, if this reservoir is sufficiently big relative to the number of actively infected cells.

Next, we break the balance between the influx into and the efflux from the reservoir by assuming that the outflow rate exceeds the inflow rate, by setting *k* = 5 x 10^−4^. Under these conditions, we implicitly assume that a low level of homeostatic proliferation takes place in the reservoir, at a rate *ρ* = 9 x 10^−3^ per day. We find that the evolutionary dynamics are even further delayed (*Fig 3C*). In particular, there is a prolonged period of time at the start of the infection in which the founder strain dominates the population and all other strains remain at very low frequency. Again, the presence of the reservoir increases the mean generation time of the virus, although this mean generation time can no longer easily be calculated due to propagation of lineages within the reservoir through homeostatic proliferation. However, we here also observe a second way in which the reservoir affects the evolutionary dynamics. At the start of the infection, the viral population in both the active compartment and the latent reservoir consists of the founder strain. No mutations take place in the reservoir, so any mutational variation in the reservoir originates from inflow from the active compartment. Homeostatic proliferation sustains the strains already present in the reservoir, and so the reservoir functions as a memory of ancestral strains (i.e. the strains that entered the reservoir earlier in infection), making them disproportionately represented in the reservoir compared to the active compartment. Strains present in the reservoir will constantly be fed back into the active compartment via activation of latently infected cells, effectively providing these strains with a fitness advantage and reducing the rate at which other, “new”, strains grow in frequency in the active compartment. This memory function of the reservoir is illustrated by the reservoir dynamics (*Fig 3C,* right panel), which clearly lag behind those of the active compartment. Although the reservoir also functions as an archive of the initial founder strain to some extent in the absence of homeostatic proliferation (*Fig 3B*), the memory effect is much stronger if latently infected cells do proliferate at low rates (*Fig 3C*), since then both the initial strain and strains that later enter the reservoir are actively sustained within the reservoir.

In conclusion, we find two distinct mechanisms by which the latent reservoir affects the within-host evolutionary dynamics: (i) cycling through the reservoir causes a direct delay of the evolutionary dynamics because even when entry is rare, viral lineages spend a considerable amount of time in the reservoir where no mutations occur, and (ii) the reservoir can provide an archive of ancestral viral strains since they are at a higher frequency in the reservoir than in the active compartment. Homeostatic proliferation of latently infected cells greatly enhances the effect of this second mechanism.

Similar results are found when within-host evolution is neutral (*Fig 3D–3F)*. We find that the presence of a reservoir can delay the rate at which neutral mutations accumulate (compare *Fig 3D and 3E*), and that this delay increases further if latently infected cells proliferate at a low rate (*Fig 3F*). The most profound differences between the within-host selection model and the within-host neutral model are seen in the presence of homeostatic proliferation in the reservoir (*Fig 3C and 3F*). Because of the relatively fast rate at which we assume neutral mutations accumulate (*μ* = 5 x 10^−3^ to gain an extra neutral mutation in the within-host neutral model, compared to *μ* = 5 x 10^−5^ to mutate into the next strain in the within-host selection model), in the within-host neutral model we do not observe a long delay during which the founder strain dominates. However, as in the selection model, the reservoir dynamics lag behind those in the active compartment and as a result strains carrying fewer neutral mutations dominate the population for much longer than in the absence of homeostatic proliferation (*Fig 3F*).

**Fig 4:**
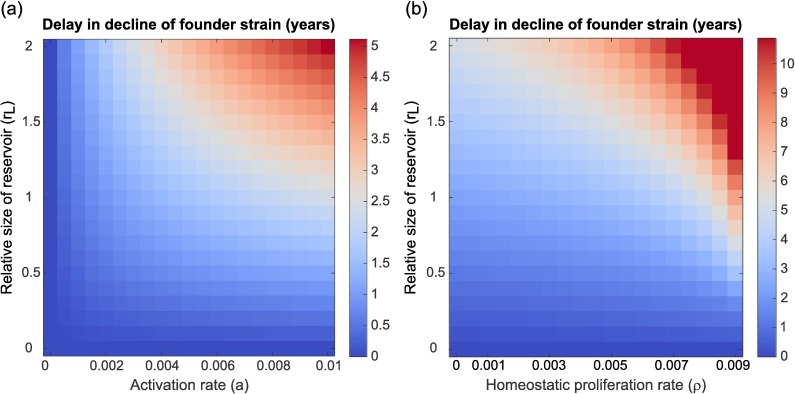
Delay in decline of the founder strain for varying reservoir parameter values in the within-host selection model. Persistence of the founder strain is defined as the time it takes for the founder strain to decline to a frequency <10% in the within-host population, and the delay in decline was calculated as the difference in persistence between the settings of interest and a control case in which the reservoir was absent. (a) Varying the activation rate *a* and the relative reservoir size *r*_*L*_ in the absence of homeostatic proliferation (*ρ = 0*, *k = r*_*L*_ *∙ a*), and (b) varying the homeostatic proliferation rate *ρ* and the relative reservoir size r_*L*_ for fixed activation rate (*a* = 0.01 per day). The homeostatic proliferation rate was varied by tuning the proportion of newly infected cells that enter the reservoir, *k*. The delay of the within-host dynamics increases with the relative size of the reservoir, and with the activation and homeostatic proliferation rate of latently infected cells. Note that the scales in panel (a) and (b) are different: the delays found in the presence homeostatic proliferation can be much larger than if latently infected cells do not proliferate. Results are shown for strains with increasing replication rates (*γ*_*1*_ = 1.0 - *γ*_*16*_ = 1.05), with the infection founded by strain 9. Similar results were found for the within-host neutral case (*S1 Fig*).

To further investigate the effect of the relative reservoir size, *r*_*L*_, the activation rate, *a*, and the homeostatic proliferation rate, *ρ*, on within-host dynamics, we calculated the delay in the evolutionary dynamics caused by the inclusion of a reservoir for different values of these parameters (*Fig 4*). Specifically, we consider the time after infection at which the frequency of the initial strain has declined to <10%, and calculate the delay as the difference between this time and the case without a latent reservoir. This metric of delay in time till decline of the initial strain was chosen to measure both the delaying and the archiving effect of the reservoir (which most profoundly affects the initial strain), with the 10% cut-off chosen arbitrarily. First, we consider a balanced case in which for every parameter combination the inflow parameter *k* is tuned such that reservoir inflow and outflow are equal (i.e. *k = a ∙ r*_*L*_), meaning there is no homeostatic proliferation in the reservoir. We find that the delay caused by including the reservoir increases with both the activation rate of latent cells and the relative size of the reservoir (*Fig 4A*). The result for the relative reservoir size can easily be understood from the intuitive argument presented above: as the relative reservoir size increases, the mean generation time of viral lineages, which is approximately equal to *1 + r*_*L*_ if *k << 1*, will increase as well. The effect of the activation rate is a bit more subtle. As the activation rate increases, the outflow of cells from the reservoir (i.e. *a ∙ r*_*L*_) rises, thus increasing the influence the reservoir has on the active compartment. However, if the outflow rate increases in the absence of homeostatic proliferation, the influx probability *k* also needs to increase to maintain the constant relative reservoir size, and an increased influx from the active compartment into the reservoir will reduce the memory effect of the reservoir. For the within-host selection model, we find here that the increase in effect of the reservoir on the active compartment outweighs the reduction in memory function caused by the effect of the active compartment on the reservoir, and the highest evolutionary delays occur when the activation rate of latently infected cells is large. The balance between these two effects is however clearly illustrated in the within-host neutral model, where we find that the within-host evolutionary dynamics are most delayed for high relative reservoir sizes but intermediate values of the activation rate *a* (*S1A Fig*).

Next, we fix the activation rate (*a* = 0.01 per day) and study the effect of changing the relative reservoir size, *r*_*L*_, and the probability that newly infected cells become latent, *k*, thus implicitly varying the homeostatic proliferation rate *ρ* (remember $\rho =a-\frac{k}{r_{l}}$). As observed above, we find that a low level of homeostatic proliferation in the reservoir increases the observed delay of the evolutionary dynamics (*Fig 4B*) since the homeostatic proliferation augments the memory function of the reservoir. Similar effects of the relative reservoir size *r*_*L*_ and homeostatic proliferation rate *ρ* on the within-host dynamics were found for the within-host neutral model (*S1B Fig*).

So far, we have considered a relatively small fitness difference of 5% between the slowest and fastest replicating strains, representing, for example, mutations towards the population consensus that generally have a small effect and which take a long time to sweep through a within-host population [48]. However, some mutations can have much larger within-host benefits and sweep through quickly. In line with these observations, in our model we find that mutations that confer a large within-host fitness benefit rapidly sweep through both the active compartment and the reservoir (*S2 Fig)*. The presence of a reservoir has very little effect on the speed of these dynamics even if homeostatic proliferation in the reservoir occurs (*S2 Fig*).

In conclusion, we have shown that the presence of a latent reservoir can drastically delay within-host evolutionary dynamics. This effect depends on the fitness effect of mutations: for neutral or small-effect mutations substantial delays are expected, but not if mutations have a moderate or large effect on within-host fitness. The delay of within-host evolutionary dynamics increases with the relative size of the reservoir, and the activation rate and homeostatic proliferation rate of latently infected cells. Our model thus predicts a negative correlation between the relative reservoir size and the within-host evolutionary rate.

### Between-host dynamics

#### Model

Next, we consider how the impact of the reservoir on within-host dynamics influences the evolution of virulence at the between-host level. To describe this between-host level, we follow Lythgoe *et al*. [20] and use a standard demographic susceptible-infected (SI) model with several types of infected individuals (see [49]). For a full description of the model, see the *Methods*. In short, we define a type-*j* infected individual as someone initially infected by strain *j*, and track *I*_*j*_(*t)*, the total number of type-*j* infected individuals in the population over time. New susceptible individuals are added to the population at rate *B* individuals per year, while all individuals have a natural mortality rate *ν* per year. Furthermore, we assume that all type-*j* infected individuals die at time *T*_*j*_ (years) after infection, which represents disease induced mortality. A full overview of the model parameters is given in *Table 1.*

The within-host model is nested in this between-host framework via strain-specific infectivity profiles *β*_*ij*_*(τ)* [20,50]. These profiles describe the rate at which type-*j* infected individuals transmit strain *i* virus at time *τ* after infection. The strain specific infectivity profile is the product of the frequency of strain *i* in the host, *x*_*ij*_*(τ)*, which we solve from the within-host model, and a predefined total infectivity profile, *α*_*j*_*(τ)*, which is only dependent on the infecting strain, *j*. Individuals initially infected with a highly virulent strain (high set-point viral load) have a short chronic infection period but high overall infectivity during this chronic phase, whereas individuals initially infected with a low virulent strain (low set-point viral load) have a long chronic infection period with a low overall infectivity (*S3A Fig*), and consequently moderately virulent strains have the highest transmission potential [20,35,36], where this is defined as the average number of secondary infections caused by an individual infected by strain *j* during their entire infectious period in an otherwise totally susceptible population. Duration and infectivity of the acute and late phases of infection are assumed to be the same for all infections.

Since viral load has been shown to correlate with the replicative capacity of the virus as measured *in vitro*, albeit weakly [28–30], and remains approximately constant during chronic infection, in the within-host selection scenario we assume that the set-point viral load of a type-*j* infected individual is determined by the replication rate *γ*_*j*_ of the initial infecting strain. Specifically, we assume that high values of *j* represent strains with a large replicative capacity and consequently highly virulent infections, whereas low values of *j* represent strains with a small replicative capacity and therefore less virulent infections. To form a baseline expectation of the results of between-host selection, we also investigate a within-host neutral scenario in which all strains are assumed to have the same within-host replicative rate, but where we continue to assume that high values of *j* represent very virulent strains and low values of *j* represent less virulent strains. Thus, even though in this scenario mutations are neutral at the within-host level, they are not neutral at the between-host level and intermediate stains within a high transmission potential are expected to be selected.

#### Epidemiological dynamics and evolution of virulence

**Fig 5:**
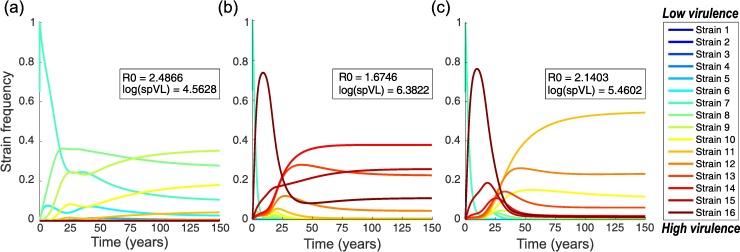
Dynamics of the between-host model. The relative prevalence of infections initiated by different viral strains, *I*_*j*_*(t)*, is shown. The insets show the basic reproduction number *R*_*0*_ and average set-point viral load (spVL) at the end of the numerical integration. Panels (a) and (b) show baseline expectations for the case without a latent reservoir, comparing a scenario in which within-host evolution is neutral to the within-host selection model. Panel (c) illustrates the effect of adding a latent reservoir. (a) No selection within-host with no reservoir. All strains have equal within-host fitness (*γ*_*1*_ = *γ*_*16*_ = 1.0), but do differ in spVL and associated virulence and duration of the infection. Moderately virulent strains are selected, which have optimal transmission potential. (b) Within-host selection model with no reservoir; strains have linearly increasing within-host replication rates (between *γ*_*1*_ = 1.0 and *γ*_*16*_ = 1.05) and increasing virulence. We clearly see the effects of short-sighted evolution: more virulent strains dominate the population, even though this reduces the population level fitness (*R*_*0*_) of the infection. (c) Within-host selection model with a reservoir; strains have increasing fitness and there is a relatively large reservoir (*r*_*L*_ = 0.5) with a low level of homeostatic proliferation (*a* = 0.01 per day, *k* = 5 x 10^−4^, *ρ =* 9 x 10^−3^ per day, settings as in *Fig 3C*). The presence of a latent reservoir delays within-host dynamics, resulting in the dominance of less virulent strains at the population level, and hence reduces the effects of short-sighted within-host evolution.

We again consider a case with *n* = 16 viral strains, and in all simulations a population of *N* = 10,000 individuals was initialised with one individual infected with a moderately virulent strain (strain 7). The nested model was numerically solved to find the epidemiological and evolutionary dynamics. Example dynamics are shown in *Fig 5*. Equilibrium population sizes vary between *N =* 1,150 individuals (infection with high virulence) and *N* = 2,150 individuals (moderately virulent infection). In the absence of a reservoir, and where all strains have the same within-host fitness, as expected the strain with maximum transmission potential and moderate virulence is selected (e.g. strain 9 in our 16-strain scenario; *Fig 5A*). The basic reproduction number, *R*_*0*_, of the viral population, which is defined as the average number of new infections a single infected individual would cause in a fully susceptible population over the course of its infection, is optimal and the average set-point viral load is close to the value observed in populations of untreated individuals: log(spVL) = 4.5 [35]. However, if strains have different within-host fitnesses, rapidly replicating high virulence strains quickly dominate within hosts and are transmitted to the next host. Consequently, in the absence of a reservoir within hosts, new infections are dominated by highly virulent strains and we observe an associated drop in the basic reproduction number *R*_*0*_ and increase in the average set-point viral load in the host population (inset in *Fig 5B*). Thus, these results illustrate how short-sighted within-host selection for high replicative capacity dominates the evolutionary process at the between-host level (see also [20]).

In contrast, when a latent reservoir is included in the model we see the dominance of intermediately virulent strains at the population level, even when there is selection for more virulent strains within individuals (*Fig 5C*). Although the basic reproduction number and the average set-point viral load are not optimal in this population, the effect of the reservoir dynamics on evolution at the population level is substantial: the mere presence of a latent reservoir causes a drop of 1.0 log in the average set-point viral load. Hence, in this example the latent reservoir has a major effect on the evolution of HIV at the population level and could to some extent explain why the virus has not evolved to be highly virulent.

This result can be understood in terms of the timescales of within-host evolution on the one hand, and transmission and duration of the infection on the other hand. The total duration of a type-*j* infection, *T*_*j*_, sets a time limit on when within-host evolution and transmission to susceptibles can occur. In the presence of a reservoir the initial strain dominates the within-host viral population for a larger proportion of this infectious period than when the reservoir is absent (*S3A–S3C Fig*), thus increasing the probability that the infecting strain, or strains with a similar virulence, are transmitted, which in turn enables the between-host adaptation of the virus.

As with the within-host dynamics, model outcomes depend strongly on the choice of parameters, particularly the relative reservoir size, the activation rate of latently infected cells, and the degree of homeostatic proliferation of latently infected cells (*S4 Fig*). Importantly, the results of this analysis are very similar to the within-host results shown in *Fig 4*. The larger the within-host delay of the evolutionary dynamics, the higher the *R*_*0*_ and the lower the average spVL in the population-level model. This is an intuitive result, since delay of the within-host evolutionary dynamics results in a longer period of time in which the founder strain can be transmitted, thus providing the virus with a mechanism to “escape” the effects of short-sighted within-host evolution.

Because of the strong dependence of our model results on the relative reservoir size *r*_*L*_, we revisited the simplifying assumption that this relative reservoir size remains constant over the entire course of an infection. We based this assumption on *in vivo* observations showing that the reservoir fills up quickly at the start of infection [3,4]. To check that our model parameters are consistent with these observations, we developed a simplified population dynamical version of our model that describes the number of actively and latently infected cells during the acute, chronic and late phases of the infection (*S1 Text*). Even though the probability of reservoir entry, *k*, and the activation rate of latently infected cells, *a*, are assumed to be low, we find that the size of the reservoir quickly increases after initial infection and can stabilise early in the chronic phase (*S5 Fig*). The results of this population dynamical model also illustrate that we should expect the relative reservoir size to be much smaller during the acute and late phases of infection because the viral load, and hence the number of actively infected cells, will typically be much higher during these phases than during the chronic phase, due to the absence or failure of immune responses mounted by the host. To examine the effect of smaller relative reservoir sizes during the acute and late phases of infection, we repeated our analyses under the conservative assumption that the latent reservoir only impacts the evolution of the active compartment during chronic infection, while the within-host evolution during the acute and late phases of infection is only determined by the dynamics of the active compartment. The results were almost identical those of the original model (*S3D* and *S6 Figs*), indicating that our conclusions do not depend on the simplifying assumption of a constant relative reservoir size throughout the entire course of infection. This is because, even in the absence of a reservoir, little evolutionary change happens during the first three months (i.e. the acute phase) of the infection (*Fig 3*), and similarly little evolution takes place in the final nine months, or the late phase, of most infections (*S3 Fig*).

## Discussion

We have investigated how the presence of a latent reservoir affects the evolutionary dynamics of HIV within individuals and at the population level. Surprisingly, we find that the latent reservoir can cause a significant delay to the within-host evolutionary process. Furthermore, if within-host evolution is sufficiently delayed the virus can evolve intermediate levels of virulence which optimise transmission at the population level. This is in contrast to the situation in which a within-host reservoir is absent and where high virulence strains are expected to dominate. Hence, the delay in within-host evolution can prevent the short-sighted evolution of very high levels of virulence.

It is important to note that these between-host effects only occur if the latent reservoir is relatively large compared to the active compartment. In the lower ranges of our estimates for the relative reservoir size (e.g. *r*_*L*_ ≈ 0.1), almost no effect of the reservoir on within-host evolutionary dynamics was found. For these conditions, the preferential transmission of strains similar to the ancestral virus, stored in long-lived memory cells, might provide an alternative explanation to the observed population level adaptation of virulence [21]. This is not an unreasonable hypothesis: ancestral strains are, by definition, transmissible, and during the course of a single infection there will not be selection to maintain transmissibility *per se*. There is now a growing body of indirect evidence that ancestral virus might be preferentially transmitted, including work on individual transmission pairs [51,52], a large cohort study [53], and phylogenetic studies [16,18,19].

Our model can be extended to investigate the population level effects of preferential transmission of ancestral virus. As a case study, we incorporated this mechanism into our model by assuming that, during the course of an infection, the virus acquires additional mutations that lower its relative transmissibility per transmission event compared to strains carrying fewer of these mutations, but which are neutral at the within-host level and do not affect virulence (*S2 Text*). We then find that inclusion of preferential transmission of ancestral virus can result in the dominance of strains with intermediate virulence that maximise the population level reproduction number, *R*_*0*_, of the infection, even for cases where high virulence strains dominate in the absence of preferential transmission (*S7 Fig*). As might be expected, however, the effect of this preferential transmission depends on the strength of the preference and the rate at which transmissibility-lowering mutations are acquired, and we only find population level adaptation of the virus if preferential transmission is sufficiently strong (*S8 Fig*). Although this model provides a proof of concept that preferential transmission of ancestral virus can lead to population level adaptation of the virulence, more biologically realistic models should be developed to further study this mechanism.

Because both our within-host and between-host results are very sensitive to the relative size of the reservoir compared to the number of infected actively replicating cells, our model produces testable predictions for the effect of the relative reservoir size on the within-host evolutionary dynamics of the virus. The larger the reservoir compared to the active compartment, the more delayed within-host evolutionary dynamics are expected to be, ranging from almost no delay in individuals with a small relative reservoir size (lower bounds of our *r*_*L*_ estimates) to a delay of multiple years in individuals in whom the reservoir is large relative to the number of actively infected cells (higher bounds of our *r*_*L*_ estimates). In particular, this might explain the low rates of evolution in natural controllers of HIV infection who maintain very low plasma HIV RNA levels, and in whom we therefore speculate that the relative size of the reservoir compared to the active compartment is large. In these patients there is growing evidence for low levels of ongoing viral replication [54–56] and the evolutionary rate has been estimated to be 3–4 times lower than in non-controllers [55]. Furthermore, in another study a negative correlation between set-point viral load and evolutionary rate has been described [57], a trend that we would predict if a high set point viral load is indicative of a small relative reservoir size. Although these data support the hypothesis that a relatively large latent reservoir delays within-host evolutionary dynamics, caution is needed in interpreting these and other results since confounding factors, such as APOBEC induced mutations, also affect both set-point viral load and rates of evolution [58].

Quantifying the number of latently infected cells containing viable provirus in HIV patients is a major challenge. Empirical estimates of the size of the latent reservoir within patients on ART can vary 2 logs in magnitude depending on the experimental method used [59]. Viral outgrowth assays tend to underestimate the reservoir size, while PCR based methods tend to lead to an overestimation [60]. We have hoped to avoid some of these biases by estimating a *relative* reservoir size compared to the number of actively infected cells, which should be relatively insensitive to errors in estimates of the absolute number of infected active and latent cells, as long as both are biased in the same way. Using both direct and indirect methods we found that the relative size of the latent reservoir can vary enormously among patients, ranging from 0.06 to 3.1 latently infected cells per infected active cell. At this moment it is unclear to what extent this variation is caused by differences in the experimental methods used or is true variation between the patients themselves. Other studies have suggested that the relative size of the reservoir is much smaller than we have estimated here because most viral DNA in the reservoir is not viable (e.g. [15]). However, recent evidence suggests that the same is true of viral DNA in actively replicating cells [58], leading us to conclude that our estimates of the relative reservoir size are probably in the right ball park. Furthermore, although at first sight our estimates for the relative reservoir size might appear high, it is important to recognise that they represent a “snapshot” of viral dynamics. Although many active CD4+ T cells are infected over the course of an HIV infection, the total number of actively infected cells at any given point is small [1]. Thus, the ratio between the numbers of latently and actively infected cells at any given time point might be larger than intuitively expected.

As well as relative reservoir size, the rate at which the reservoir is replenished and depleted, and hence the (assumed) homeostatic proliferation rate in the reservoir, is another important determinant of within-host dynamics. There is a growing body of evidence suggesting that latent reservoirs are maintained by homeostatic proliferation in patients on antiretroviral therapy. Modelling studies have suggested that homeostatic proliferation must occur to maintain stable reservoir sizes under therapy [61,62], and in support of this Chomont *et al*. [26] found direct evidence of ongoing homeostatic proliferation in a subset of infected memory CD4+ T cells. More recent studies of HIV DNA integration sites at single-cell level [63,64] found that multiple cells carry identical proviral sequences at the same integration site, suggesting that up to 40% of cells in the latent reservoir have arisen from clonal expansion [63]. We should however be careful in interpreting these results, since the clonally expanded proviruses were not tested for replication competency. In a different study Cohn *et al*. [65] found that although the majority of integrated proviruses resided in clonally expanded T cells, all of the 75 proviruses found in these clones were defective. However, since in our model we only assume a very low level of proliferation of latently infected cells, if at all, these results do not directly contradict our assumptions.

We find that the within-host evolutionary dynamics of HIV can be severely delayed by the presence of a latent reservoir if mutations are neutral within-host or have a small within-host fitness effect. Some mutations might however confer a larger within-host fitness benefit, as has been suggested for some immune escape mutations [66–68], although other studies have found that immune escape might be relatively slow, especially during the chronic phase of infection [66,69,70]. For mutations that have a large effect on within-host fitness, we predict that the presence of the reservoir will have very little effect on within-host evolutionary dynamics because the fittest variant will quickly sweep the active compartment and the reservoir. However, since recombination happens very frequently during HIV replication [48,71,72] we still expect to see the predicted effect of the latent reservoir when considering neutral mutations or mutations with small within-host fitness effects.

When modelling the evolution of virulence at the population level, we have confined ourselves to considering viral factors, and not host factors or host-virus interactions, which are likely to be mediated through the host immune response. The high heritability of HIV set-point viral load [21] suggests that viral factors are important determinants of virulence, although as yet we do not know what these factors are, and this is the subject of ongoing research. Since the human population is very heterogeneous (e.g. with regard to immune responses) and this might impact epidemiological and evolutionary dynamics [39], an important next step will be to determine how the incorporation of host heterogeneity affects our conclusions.

Because of the problems they pose to eradication of viral infection, latent HIV reservoirs have mainly been studied in the context of antiretroviral treatment [7,8]. Here, however, we have investigated how reservoir dynamics influence the evolutionary dynamics of HIV in untreated individuals with ongoing active viral replication. Overall, our models have shown that latently infected cells can have a major impact on the evolution of the virus. The latent reservoir plays a role in the determination of the within-host evolutionary rate and the outcome of population level evolution of virulence. Therefore, latently infected cells should be considered if we want to understand the evolutionary dynamics of HIV at the within- and between-host levels.

## Methods

We developed a multilevel mathematical model that nests the within-host evolutionary dynamics of HIV in a between-host epidemiological model of HIV using a time-since-infection framework. At the between-host level, we follow Lythgoe *et al*. [20] and use a standard multitype epidemic model [49]. At the within-host level, we use two coupled quasi-species equations [40] to describe the frequency distribution of different viral strains in actively infected cells and the latent reservoir. We model strain frequencies at the within-host level, rather than the total number of virions (or infected cells) of each strain, since tractable mathematical models of HIV infection that can reproduce the observed ranges of profiles of infection among patients (such as differences in spVL and duration of infection) do not currently exist [41]. In our between-host model, we therefore use known associations between spVL, transmissibility and duration of infection to characterise the overall infectivity profile, *α*_*j*_*(τ)*, of a host initially infected by strain *j* at time *τ* after infection. Using our within-host model, we separately model the frequency of strains *i* within a host infected by strain *j*, *x*_*ij*_*(τ)*, assuming an infinite population size, and then couple the between-host and within-host levels via the strain specific infectivity profile *β*_*ij*_*(τ)*, which is a combination of *x*_*ij*_*(τ)* and *α*_*j*_*(τ)* and describes the infectivity of strain *i* in a host originally infected with strain *j* at time *τ* after infection [20,50]. All models were implemented in Matlab, version R2013a.

### Within-host model

Within a host we consider two compartments: an active compartment and a latent reservoir. The active compartment describes the infection of active CD4+ T cells. In this compartment, viral replication (i.e. new infections) and mutation take place. A small fraction, *k*, of the newly infected cells enter the reservoir compartment as long-lived latently infected cells. These cells are reactivated at a rate *a* per day. An overview of the model structure is shown in *Fig 1.*

Consider *n* different viral strains that can differ in their replication rate, *γ*_*i*_ *(i =* 1, 2, …, *n)*. Let *x*_*i*_ be the frequency of strain *i* in the active compartment, and *y*_*i*_ its frequency in the latent reservoir. Mutations are described by a mutation matrix *M = (m*_*ij*_*)*, where *m*_*ij*_ is the probability that strain *j* mutates into strain *i* during replication. The dynamics of the system are described by the following differential equations:

$$\left\{ \begin{array}{l} \frac{dx_{i}}{dt}=(1-k)\sum_{j=1}^{n}m_{ij}\gamma_{j}x_{j}+ar_{L}y_{i}-x_{i}((1-k)\sum_{j=1}^{n}\gamma_{j}x_{j}+ar_{L}\sum_{j=1}^{n}y_{j})\\ \frac{dy_{i}}{dt}=\frac{k}{r_{L}}\sum_{j=1}^{n}m_{ij}\gamma_{j}x_{j}-ay_{i}-y_{i}(\frac{k}{r_{L}}\sum_{j=1}^{n}\gamma_{j}x_{j}-a\sum_{j=1}^{n}y_{j}), \end{array}\right.$$ (1)

or in matrix-vector notation

$$\left\{ \begin{array}{l} \frac{dx}{dt}=(1-k)Qx+ar_{L}y-x((1-k)\overset{-}{\gamma }+ar_{L})\\ \frac{dy}{dt}=\frac{k}{r_{L}}Qx-ay-y(\frac{k}{r_{L}}\overset{-}{\gamma }-a), \end{array}\right.$$ (2)

where ***x*** *= (x*_*1*_, *x*_*2*_, *…*, *x*_*n*_*)*^*T*^, ***y*** *= (y*_*1*_, *y*_*2*_, *…*, *y*_*n*_*)*^*T*^, *Q = (q*_*ij*_*) = (m*_*ij*_ *γ*_*j*_*)* is the replication-mutation matrix, and $\overset{-}{\gamma }=\sum_{j=1}^{n}\gamma_{j}x_{j}$ is the total replication rate of the virus. The last term in all of these equations represents a chemostat-assumption, which assures that the frequencies *x*_*j*_ and *y*_*j*_ add up to one at all times [40]. This chemostat-assumption is equivalent to assuming that the death rate of cells within a given compartment is the same for all strains.

Note that *Eq 1*introduces an additional parameter, the relative reservoir size *r*_*L*_. Since we describe strain frequencies, rather than absolute numbers of infected cells, this parameter is needed to account for a potential difference in size between the two connected compartments. Let *A* be the total number of infected active CD4+ T cells, and *L* the total number of latently infected memory CD4+ T cells. Then, the number of memory CD4+ T cells latently infected with strain *i* is given by *y*_*i*_ *L*. Since these cells are reactivated at a rate *a* per day, the total rate of re-entry of strain *i* into the active compartment is *ay*_*i*_*L* cells per day. The contribution of these reactivated cells to the change in frequency of strain *i* in the active compartment is given by

$$\Delta x_{i}(a)=\frac{ay_{i}L}{A}$$ (3)

since the size of the active compartment is *A* cells. A similar argument can be made to describe the contribution of newly latently infected cells to the strain distribution in the reservoir, which is given by

$$\Delta y_{i}(k)=\frac{k\sum_{j=1}^{n}m_{ij}\gamma_{j}x_{j}A}{L}=\frac{A}{L}k\sum_{j=1}^{n}m_{ij}\gamma_{j}x_{j}.$$ (4)

In both cases we see that if the reservoir and the active compartment are of unequal size, this size difference has to be accounted for. Hence, we define

$$r_{L}=\frac{L}{A}$$ (5)

as the relative size of the reservoir. The use of this parameter *r*_*L*_ is also illustrated in *Fig 1*.

Note that our model implicitly includes the possibility of homeostatic proliferation of latently infected cells. Viral load stays approximately constant during chronic infection, and therefore we can expect the number of actively infected cells (i.e. the absolute size of the active compartment) to be relatively constant as well, as is also predicted by models of within-host infection dynamics (see [9] and [10] for recent reviews). In the model, we make the additional assumption that the relative size of the reservoir compared to the active compartment remains constant during chronic infection. This means that the absolute size of the reservoir must also remain the same (see *Eq 5*). Using the notation of *Eq 5*, let *A* and *L* be the absolute size of the active compartment and the reservoir, respectively. Since $\sum_{j=1}^{n}\gamma_{j}x_{j}\approx 1$ (see *Table 1*for parameter values) the total entry rate of cells entering the reservoir is approximately *k ∙ A*, while the total outflow rate of cells leaving the reservoir is *a ∙ L*. To maintain a constant reservoir size in the absence of homeostatic proliferation these entry and outflow rates must be equal, i.e. $k=a\cdot \frac{L}{A}=a\cdot r_{L}$. If however the outflow is greater than the inflow (i.e. $a>\frac{k}{r_{L}}$), then homeostatic proliferation of latently infected cells is implicitly assumed to take place so as to maintain the reservoir size, at a rate

$$\rho =a-\frac{k}{r_{L}}.$$ (6)

Since new HIV infections are generally established by a single viral genotype [45–47], we assume that all within-host processes are started with only a single strain present at time *τ = 0*. It has been shown that the reservoir of latently infected cells is established very quickly, as early as 10 days after infection [3,4]. We incorporate this into our model by assuming that the initial strain is immediately present in both the active compartment and the latent reservoir. From these initial conditions, *Eq 2*is numerically integrated to calculate *x*_*ij*_*(τ)* and *y*_*ij*_*(τ)*, the frequency of strain *i* in the active compartment and the reservoir at time *τ* after infection in an individual originally infected with strain *j* at time *τ = 0*. Note that a constraint of using the time-since-infection framework is the assumption that no superinfection takes place. Numerical integration was done with Matlab’s built-in differential equation solver ode45, using default settings.

Then, following Lythgoe *et al*. [20] we define the strain specific infectivity profile

$$\beta_{ij}(\tau )=\alpha_{j}(\tau )x_{ij}(\tau ).$$ (7)

Here, *α*_*j*_*(τ)* is a predefined overall infectivity profile of infections established by strain *j*. We assume that the infectivity profile *α*_*j*_*(τ)* consists of three stages: acute infection, a chronic phase and a late phase. The durations of the acute and late phases are assumed to be equal for all infections, as is the infectivity during these phases. The duration and infectivity of the chronic phase, however, are assumed to be functions of spVL: infections with higher spVLs have shorter chronic phases, and higher infectivities during this chronic phase, than infections with lower spVLs (illustrated in *S3 Fig*, see [20,35,36] for details and parameter estimates). Because the duration of the chronic phase depends on the spVL, the total duration of the infection, *T*_*j*_, will also be shorter for more virulent strains (i.e. those with a higher spVL) [20,35]. Viral load is relatively stable during chronic infection, even though a large amount of within-host evolution takes place during this period. We therefore assume that the spVL in turn is determined by the infecting strain. Since spVL correlates with replicative capacity of the virus [28–30], we furthermore assume that strains with higher replication rates result in infections with higher spVLs [20].

### Between-host model

Following Lythgoe *et al*. [20], we use the strain specific infectivity profiles described by *Eq 7*to nest the within-host dynamics into a multitype epidemiological model to describe the between-host dynamics [49]. Here we will provide a short description of the model, see [20] and [49] for the full derivation.

We use a susceptible-infected model with demography. Let *S(t)* and *I(t)* be the number of susceptible and infected individuals at time *t*, respectively, and let *N(t) = S(t) + I(t)* be the total number of individuals in the population. Let *I*_*i*_*(t)* be the number of type-*i* infected individuals, i.e. individuals initially infected with strain *i*. Then, $I(t)=\sum_{i=1}^{n}I_{i}$. Assume that all individuals die at a natural death rate *ν*, and furthermore that type-*i* infected individuals die at time *T*_*i*_ after initial infection if they have not succumbed to natural mortality. Let *B* be the rate at which new susceptible individuals enter the population. Finally, let *H*_*i*_*(t)* denote the incidence of type-*i* infections, i.e. the rate at which new type-*i* infections occur. Assuming random mixing of the population, the epidemiological dynamics are then described by

$$H_{i}(t)=\frac{S(t)}{N(t)}\sum_{j=1}^{n}\int_{0}^{T_{j}}\beta_{ij}(\tau )H_{j}(t-\tau ){\text{e}}^{-\nu \tau }d\tau ,$$ (8)

$$S(t)=N(t)-\sum_{i=1}^{n}\int_{0}^{T_{i}}H_{i}(t-\tau ){\text{e}}^{-\nu \tau }d\tau ,$$ (9)

$$\frac{dN}{dt}=B-\nu N(t)-\sum_{i=1}^{n}H_{i}(t-T_{i}){\text{e}}^{-\nu T_{i}},$$ (10)

$$I_{i}(t)=\int_{0}^{T_{i}}H_{i}(t-\tau ){\text{e}}^{-\nu \tau }d\tau .$$ (11)

These equations were numerically integrated using Matlab, using Matlab’s function trapz to numerically solve the integrals and the forward Euler method to solve the differential equations (timestep *dt* = 0.01 year).

The next-generation matrix $K=(k_{ij})=(\int_{0}^{T_{j}}\beta_{ij}(\tau ){\text{e}}^{-\nu \tau }d\tau )$ can also easily be calculated. This next-generation matrix can be used to quickly find the equilibrium of the model without numerically solving *Eqs 8–11*. Namely, at equilibrium the basic reproduction number *R*_*0*_ of the infection is equal to the dominant eigenvalue of the next-generation matrix *K*, while the corresponding eigenvector (normalised to have components summing up to one) gives the relative incidence $\left({\overset{-}{H}}_{i}^{*}\right)$ [20,49]. *R*_*0*_ represents the average number of new infections one infected individual would cause in a fully susceptible population during its lifetime. At equilibrium, however, the number of infected individuals is constant over time and hence the number of new cases arising from a single infection should be equal to one. Therefore, at equilibrium

$$\frac{S^{*}}{N^{*}}=\frac{1}{R_{0}}$$ (12)

where the star denotes equilibrium values. Setting *Eqs 8–11*to equilibrium and using *Eq 12*we can now directly derive

$$I_{i}^{*}=\frac{B(R_{0}-1){\overset{-}{H}}_{i}^{*}(1-{\text{e}}^{-\nu T_{i}})}{\nu (R_{0}-\sum_{j=1}^{n}{\overset{-}{H}}_{i}^{*}{\text{e}}^{-\nu T_{i}})},$$ (13)

and similar expressions for *H*_i_*, S* and N* (see [20]). Since *R*_*0*_ and $\left({\overset{-}{H}}_{i}^{*}\right)$ can be found from the next-generation matrix *K*, all equilibrium values can thus be directly calculated from the next-generation matrix.

### Estimation of the relative reservoir size during chronic infection

To estimate the relative reservoir size *r*_*L*_, two data sources were used: (i) a direct report on the number of active and latent CD4+ T cells with integrated HIV-1 DNA in several chronic phase patients [1], and (ii) several studies reporting HIV-1 DNA levels pre- and during anti-retroviral treatment in patients starting treatment during chronic phase [22–26]. Chun *et al*. [1] report the concentration of cells with integrated HIV-1 DNA and the percentage of total cells that are latent. Then, the relative reservoir size can be directly calculated as

$$r_{L}=\frac{{\left[HIV_{DNA}\right]}_{l}(1-P_{a})}{{\left[HIV_{DNA}\right]}_{a}P_{a}}$$ (14)

Here, *P*_*a*_ denotes the percentage of CD4+ T cells that are activated (HLA DR+), and *[HIV*_*DNA*_*]*_*a*_ and *[HIV*_*DNA*_*]*_*l*_ are the concentrations of HIV DNA positive active and latent cells (per 10^6^ active/latent cells), respectively.

For the second set of studies, note that if treatment is effective, all HIV DNA measured during treatment can be assumed to be integrated in long-lived memory CD4+ cells [26]. If we furthermore assume that reservoir size does not decrease drastically with treatment (which is in agreement with the very long half-lives found for the reservoir [5]), the HIV DNA levels during treatment represent the size of the reservoir before treatment was initiated. Pre-treatment HIV DNA levels, however, represent the size of the total infected population (i.e. actively *and* latently infected cells). Using these proxies we can calculate the relative reservoir size as

$$r_{L}=\frac{{\left[HIV_{DNA}\right]}_{t}}{{\left[HIV_{DNA}\right]}_{n}-{\left[HIV_{DNA}\right]}_{t}},$$ (15)

where *[HIV*_*DNA*_*]*_*t*_ and *[HIV*_*DNA*_*]*_*n*_ denote the HIV DNA-level during treatment and pre-treatment (naive), respectively.

Note that we do not incorporate the fraction of defective integrated proviruses into our indirect method. Approximately 88% of integrated proviruses in resting CD4+ T cells in patients on ART are completely replication incompetent because of genetic defects [60]. However, since most of these mutations are induced pre-integration (either due to reverse transcriptase infidelity or host cell APOBEC activity), we should expect to find defective integrated proviruses in activated CD4+ T cells as well. Indeed, it was recently shown that a substantial proportion of HIV DNA sequences isolated from peripheral blood mononuclear cells from treatment-naïve patients contain premature stop codons [58]. If mutations in the HIV sequence happen at equal rate during infection of activated and resting CD4+ T cells, and if resting CD4+ cells infected with defective virus live the same amount of time and proliferate at the same rate as those latently infected with non-defective virus (an assumption that has however recently been challenged [65]), the fraction of cells infected with replication competent proviruses should be similar in both compartments. Hence, the ratio of replication competent HIV DNA levels does not change and our estimate for the relative reservoir size is unaffected.

## Supporting Information

S1 Text A population dynamical model of actively and latently infected cell dynamics during different stages of the infection. (PDF)

S2 Text Modelling preferential transmission of ancestral virus. (PDF)

S1 Table Full data used to estimate the relative reservoir size *r*_*L*_, per patient and per study. (PDF)

S1 Fig Delay in decline of the founder strain for varying reservoir parameter values in the within-host neutral model. The time until decline is defined as the time it takes for the initial strain to decline to a frequency <10% in the within-host population, and delay was calculated as the difference in time until decline between the settings of interest and a control case in which the reservoir was absent. All strains have equal within-host replication rates, and parameter settings were as described in *Table 1*. (a) Varying the activation rate *a* and the relative reservoir size *r*_*L*_ in the absence of homeostatic proliferation (*ρ* = 0, *k = r*_*L*_ *∙ a*), and (b) varying the homeostatic proliferation rate *ρ* and the relative reservoir size *r*_*L*_ for fixed activation rate (*a* = 0.01 per day). Note the different scales in (a) and (b): the maximal delays found in the presence of homeostatic proliferation are much larger than the delays in the absence of homeostatic proliferation. Homeostatic proliferation rate was varied by tuning the proportion of newly infected cells that enter the reservoir, *k*. In general, these results resemble the results found for the within-host selection model (*Fig 4*): the delay of the within-host dynamics increases with the relative size of the reservoir, and with the activation and homeostatic proliferation rate of latently infected cells. For large reservoir sizes (*r*_*L*_ > *1*) in the absence of homeostatic proliferation (*S1A Fig*), however, we find that the delay is maximised for smaller values of the activation rate *a*. This is due to the no-homeostatic proliferation assumption, which dictates that for smaller activation rates *a* the probability of cells entering the reservoir is also smaller (remember *k = r*_*L*_ *∙ a*), thus reducing the effect of within-host replication and mutation on the strain distribution in the reservoir.
(EPS)

S2 Fig **Within-host evolutionary dynamics if within-host fitness differences are large,** (a) in the absence of a reservoir (*k = a = 0*), (b) in the presence of a reservoir, but without homeostatic proliferation in the reservoir (*r*_*L*_ = 0.5, *k* = 5 x 10^−3^, *a* = 0.01 per day, *ρ* = 0 per day), and (c) in the presence of a reservoir, with a low level of homeostatic proliferation (*r*_*L*_ = 0.5, *k* = 5 x 10^−4^, *a* = 0.01 per day, *ρ* = 9 x 10^−3^ per day). Strains have linearly increasing replication rates between *γ*_*1*_ = 1.0 and *γ*_*16*_ = 1.2 and the infection is initiated with strain 9. All other parameter values are as stated in *Table 1*. Both in the absence and the presence of a reservoir the fittest strain (strain 16) rapidly sweeps the active compartment and the reservoir if present. The latent reservoir hence has little effect on the within-host evolutionary dynamics (compare these dynamics to the case with small within-host fitness differences, Fig 3).
(EPS)

S3 Fig Predefined infectivity profiles *α*_*j*_*(τ)*, and within-host evolutionary dynamics without and with a reservoir for infections initiated by strains differing in within-host fitness. Strains differ in set-point viral load, with log(spVL) linearly increasing from log(spVL) = 2 for infections initiated by strain 1 to log(spVL) = 7 for infections initiated by strain 16. For panels (b-d) strains were furthermore assumed to have linearly increasing within-host replication rates between *γ*_*1*_ = 1.0 and *γ*_*16*_ = 1.05. Plots were made for strains varying from low (strain 3) to high (strain 15) set-point viral load. (a) Predefined infectivity profiles *α*_*j*_*(τ)*, which describe the duration of the infection, as well as the infectivity during the acute, chronic and late phase of the infection [20]. Infectivity and duration of the acute and late phase are the same for all infections, but the duration and infectivity of the chronic phase depend on the set-point viral load of the infection, which is determined by the strain that initiated the infection. In the within-host selection model the spVL depends in turn on the within-host fitness of the initial strain. (b) Within-host evolutionary dynamics in the absence of a latent reservoir (*k = a = 0*). Vertical lines indicate the maximal duration of the infection, *T*_*j*_. (c) Within-host evolutionary dynamics in the presence of a latent reservoir, with a low level of homeostatic proliferation in this reservoir (*r*_*L*_ = 0.5, *k* = 5 x 10^−4^, *a* = 0.01 per day, *ρ* = 9 x 10^−3^ per day). All other parameter values are as stated in *Table 1*. Especially for the strains with high within-host replication rate and hence high virulence, we see that the addition of a reservoir delays the within-host dynamics to such an extent that for most of the duration of the infection, the initial strain dominates the within-host viral population at high frequency. (d) Within-host evolutionary dynamics under the conservative assumption that the active compartment is unaffected by the reservoir during the acute and late phases of infection. Parameter values were set as in panel (c), except the reservoir is assumed to fill up instantaneously at the end, rather than at the beginning, of the acute phase of infection. The activation rate *a* was set to 0 during the acute and the late stage of infection meaning that the reservoir could not influence the dynamics in the active compartment. Note that since the dynamics of this conservative model differ for each phase of the infection, no dynamics can be shown for this model after the end of the infection, *T*_*j*_. The results for the conservative model are very similar to the results of the original model (panel (c)), showing that the results are insensitive to the simplifying assumption of a constant relative reservoir size for the entire duration of the infection.
(EPS)

S4 Fig Basic reproduction number *R*_*0*_ and average set-point viral load predicted by the between-host model for varying reservoir parameter values. (a) Varying the activation rate *a* and the relative reservoir size *r*_*L*_ in the absence of homeostatic proliferation (*ρ* = 0, *k = r*_*L*_ *∙ a*), and (b) varying the homeostatic proliferation rate *ρ* and the relative reservoir size *r*_*L*_ for fixed activation rate (*a* = 0.01 per day). Homeostatic proliferation rate was varied by tuning the proportion of newly infected cells that enter the reservoir, *k*. Note the resemblance to *Fig 4*. High population level fitness of the virus (i.e. high *R*_*0*_ and intermediate set-point viral loads) is found precisely when the within-host dynamics are delayed.
(EPS)

S5 FigDynamics of the number of actively and latently infected cells, and the relative reservoir size during the acute, chronic and late phase of infection.A simplified population dynamical version of our model was developed to investigate the initial filling up of the reservoir, and the relative reservoir size at the different phases of infection (*S1 Text*). This population dynamical model was numerically solved starting from a single actively infected cell, no latently infected cells and a susceptible cell population at equilibrium ($W=\frac{\sigma }{d}$, see *S1 Text*), to find (a) the number of actively infected cells *X*, (b) the number of latently infected cells *Y*, and (c) the relative reservoir size *r*_*L*_. The infection is assumed to last for 5 years (1825 days), with the acute phase lasting 3 months (i.e. *τ* ≤ 120 days) and the late phase lasting 9 months (i.e. *τ* ≥ 1555 days). Results are shown for a case with a low level of homeostatic proliferation in the reservoir, corresponding to the parameters in *Figs 3C* and *5C* (*a* = 0.01 per day, *k* = 5 x 10^−4^, *ρ =* 9 x 10^−3^ per day). We set the entry rate of new susceptible cells *σ* = 10^7^ cells per day. Susceptible cells die at a rate *d* = 0.5 per day [17], the basic death rate of actively infected cells *δ*_*X*_ = 1 [42], latently infected cells die at a rate *δ*_*Y*_ = 0.001 per day [9,73], and the per capita infectivity of infected cells, *β* = 2.5 x 10^−7^ such that the within-host $R_{0}=\frac{\beta \sigma }{d\delta_{X}}=5$ newly infected cells per actively infected cell per day [74]. During the chronic phase of infection the death rate of actively infected cells is increased by *δ*_*CTL*_ to simulate killing by the host’s immune system, and results are shown for *δ*_*CTL*_ = 1 (blue line), *δ*_*CTL*_ = 2 (magenta line), *δ*_*CTL*_ = 3 (red line), and *δ*_*CTL*_ = 3.5 (orange line). Note that results for *δ*_*CTL*_ ≥ 4 cannot be obtained because that would reduce the within-host *R*_*0*_ of the infection below 1. Both the number of actively and latently infected cells increases quickly during the acute phase of the infection. When the number of actively infected cells drops at the acute-chronic transition, the relative reservoir size suddenly increases and can quickly stabilise (e.g. red line, *δ*_*CTL*_ = 3). This result does however depend on the strength of the immune response (S1 *Text*). For all choices of the parameter value of *δ*_*CTL*_, the relative reservoir size is small during the acute and late phases of the infection due to the high number of actively infected cells during these phases.(EPS)

S6 Fig Between-host results under the conservative assumption that the latent reservoir influences the evolutionary dynamics during the chronic phase only. To investigate the impact of our assumption that the relative reservoir size is constant over the entire duration of the infection, we repeated the analyses of *Fig 5C* (panel (a)) and *S4 Fig* (panels (b) and (c)), but now assuming that the evolutionary dynamics are only influenced by the reservoir during the chronic phase of infection, while the active compartment is unaffected by the reservoir during the acute and late phases of infection. The reservoir is assumed to fill up instantaneously at the end, rather than at the beginning, of the acute phase of infection. The activation rate *a* was furthermore set to 0 during the acute and late phases of infection, ensuring that the reservoir could not influence the dynamics in the active compartment during these phases of the infection. Both the between-host dynamics (panel (a)) as well as the predicted basic reproduction number R_0_ and the average set-point viral load in the population at equilibrium for varying parameter values (panels (b) and (c)) are very similar to the results found for our original model.
(EPS)

S7 Fig Epidemiological dynamics when preferential transmission of ancestral virus is included. Relative prevalence of the different strains in the population for the case where the relative reservoir size *r*_*L*_ = 0.1 and there is a low level of homeostatic proliferation in the reservoir (*a* = 0.01 per day, *k* = 10^−4^, *ρ* = 9 x 10^−3^ per day). (a) If ancestral virus is not preferentially transmitted, the population is dominated by strains with high virulence, and consequently the basic reproduction number of the infection, *R*_*0*_, is relatively low. (b) If viral strains accumulate transmissibility-lowering mutations over time and hence ancestral virus is preferentially transmitted, viral strains with lower virulence are selected at the population level and the *R*_*0*_ of the infection is high. The transmissibility-lowering mutations are assumed to be neutral on the within-host level and independent of mutations affecting within-host fitness and/or virulence. Transmissibility-lowering mutations are acquired with probability *μ*_*θ+*_ = 5 x 10^−3^ and reverted with probability *μ*_*θ-*_ = 5 x 10^−5^ during within-host replication, and each mutation decreases the *relative* transmissibility of a strain by *θ*_*step*_ = 0.25. Note that the absolute transmissibility of the virus (i.e. the infectiousness of an infected individual) is still assumed to stay constant during the chronic stage of the infection.
(EPS)

S8 Fig Effect of preferential transmission of ancestral virus on between-host viral evolution for varying parameter settings. The basic reproduction number *R*_*0*_ and the average set-point viral load at equilibrium are plotted for different values of the effect on relative transmissibility of a single transmissibility-lowering mutation (*θ*_*step*_) and the rate at which strains acquire transmissibility-lowering mutations (*μ*_*θ+*_). Plots were made for a case with relatively small reservoir size (*r*_*L*_ *=* 0.1) and a low level of homeostatic proliferation in the reservoir (*a* = 0.01 per day, *k* = 1 x 10^−4^, *ρ* = 9 x 10^−3^ per day). All other parameters as in *Fig 5*. If both *θ*_*step*_ and *μ*_*θ+*_ are sufficiently large viral strains of intermediate virulence are selected and the virus has high population level fitness (*R*_*0*_). Furthermore, the highest population level fitness is reached if *θ*_*step*_ and *μ*_*θ+*_ are well-balanced: if transmissibility-lowering mutations accumulate faster (higher *μ*_*θ+*_) then a smaller effect per mutation (lower *θ*_*step*_) will cause a similar decline of transmissibility in time (and hence in non-founder strains) as if *μ*_*θ+*_ is smaller and *θ*_*step*_ is larger. If the decline in transmissibility is very fast (either because *θ*_*step*_ or *μ*_*θ+*_ is very large), the frequency of viruses with high transmissibility (that are similar to the founder virus) will drop too quickly to contribute significantly to transmission events, even though they have a high transmission advantage.
(EPS)
