# Supplementary material for: Effect of the Latent Reservoir on the Evolution of HIV at the Within- and Between-Host Levels
Source: PLoS Comput Biol. 2017 Jan 19;13(1):e1005228. doi: 10.1371/journal.pcbi.1005228 (PMC5245781; doi:10.1371/journal.pcbi.1005228)
Supplement: S1 Text — (PDF) [file pcbi.1005228.s001.pdf]

Effect of the latent reservoir on the evolution of HIV at the within- and between-host levels

Supporting Text S1

Hilje M. Doekes[1,2], Christophe Fraser[1,3], Katrina A. Lythgoe[1,4,*]

[1] Department of Infectious Disease Epidemiology, Imperial College London, United Kingdom.

[2] Theoretical Biology, Utrecht University, The Netherlands.

[3] Oxford Big Data Institute, Li Ka Shing Centre for Health Information and Discovery, Nuffield Department of Medicine, University of Oxford, United Kingdom.

[4] Department of Zoology, University of Oxford, United Kingdom.

* katrina.lythgoe@zoo.ox.ac.uk.

A population dynamical model of actively and latently infected cell dynamics during different stages of the infection

In the main text, we consider a system of coupled quasispecies equations to model the changing strain frequencies during the course of infection in the active compartment and the reservoir. In doing this, we make the assumption that the relative reservoir size, $r_L = \frac{L}{A}$, where L is the number of latently infected cells and A the number of actively infected cells, stays constant over the entire course of the infection. One of the requirements for this assumption is that the reservoir fills up quickly at the start of an infection, as is also observed *in vivo*. Here we present a simplified dynamical version of our model, in the absence of evolution, to test the validity of this assumption.

Let W be the number of susceptible cells, X the number of infected cells in the active compartment, and Y the number of latently infected cells in the reservoir. The equations for the population dynamical version of our model are then given by

$$\frac{dW}{dt} = \sigma - dW - \beta WX\,, \quad \text{(S1.1)}$$

$$\frac{dX}{dt} = (1-k)\beta WX - \delta(t)X + aY, \quad \text{(S1.2)}$$

$$\frac{dY}{dt} = k\beta WX + \rho Y - \delta_Y Y - aY. \quad \text{(S1.3)}$$

Here, σ is the rate at which susceptible cells enter the system, d is the death rate of susceptible cells, β is the per capita infectivity of infected cells, k is the probability that a newly infected cell becomes latently infected, $\delta(t)$ is the death rate of actively infected cells, a is the activation rate of latently

infected cells, ρ is the proliferation rate of latently infected cells, and δ_Y is the death rate of latently infected cells.

As in our between-host model, we model an infection with three stages: an initial acute phase, a chronic phase and a late phase. For consistency with our between-host model and previous observations [35], we assume that the acute phase of infection lasts 3 months and the late phase of infection lasts 9 months. We model the transition from the acute phase to the chronic phase of infection and the associated drop in viral load by assuming that after three months the death rate of actively infected cells is increased due to killing by the host's immune system, and similarly describe the transition from the chronic phase to the late phase of infection and the associated rise in viral load by the subsequent loss of these immune responses. I.e.

$$\delta(t) = \begin{cases} \delta_X & if\ \tau \leq 120\ days\ or\ \tau \geq (T-270)\ days \\ \delta_X + \delta_{CTL} & if\ 120\ days < \tau < (T-270)\ days \end{cases}, \quad \text{(S1.4)}$$

where τ is the time since infection and T is the maximal duration of the infection (ignoring natural host mortality). We set $\delta_X = 1$ per day [41] and vary δ_{CTL}. We assume $\sigma = 1 \times 10^7$ cells per day [17], $d = 0.5$ per day [17], and $\delta_Y = 0.001$ per day [9,72]. We also assume that the within-host R_0 of the virus is 5 [73], giving us $\beta = \frac{R_0 d \delta_X}{\sigma} = 2.5 \times 10^{-7}$ per actively infected cell per day. For the parameters k, a, and ρ we assumed a low level of homeostatic proliferation of latently infected cells ($k = 0.0005$, $a = 0.01$ per day, $\rho = 0.009$ per day; these are the parameters used in *Fig 3c* and *Fig 5c* of the main text).

In *S5 Fig* we show the results of numerically solving equations (S1.1)-(S1.3), starting with a single actively infected cell, no latently infected cells, $\frac{\sigma}{d}$ susceptible cells, and for varying strengths of the immune response, δ_{CTL}. Both the number of actively and latently infected cells quickly increases at the start of the infection. However, since the number of actively infected cells is high the relative reservoir size during the acute phase of infection is low. As the number of actively infected cells drops due the increased effectiveness of the immune response in the chronic phase, the relative reservoir size goes up and can quickly reach equilibrium (e.g. red line in *Fig S5c*), although this takes longer the lower the strength of the immune response (δ_{CTL}). If the strength of the immune response is very big the reservoir seems to be "overfilled" and we see an overshoot in the relative reservoir size before it gradually falls to reach equilibrium levels (orange line in *Supporting Fig S5c*). During the late phase of infection, the number of actively infected cells very rapidly increases due to the failure of the host's immune system. As expected, this again causes a drop in the relative reservoir size, to a value similar to the value during the acute phase of the infection.

This dynamical model (S1.1-S1.4) provides a proof of concept for our assumption of early filling of the reservoir during the acute phase of infection (even if the probability that infected cells enter the

reservoir is very small, as we have assumed in this example) and constant r_L during chronic infection. However, it also shows that the assumption of constant r_L during the entire course of infection is more problematical. We therefore repeated our analyses with the assumption that the reservoir has no impact on the evolutionary dynamics of the active compartment during early and late stage infection (*S3d* and *S6 Figs*) and found that the effect is negligible.

We note that the results presented here are sensitive to the strength of the immune response and associated changes in viral load, and more elaborate models should be developed to more accurately describe the dynamics of actively and latently infected cells during the initial and late phases of an infection. This is however beyond the scope of this article.
