# Supplementary material for: Effect of the Latent Reservoir on the Evolution of HIV at the Within- and Between-Host Levels
Source: PLoS Comput Biol. 2017 Jan 19;13(1):e1005228. doi: 10.1371/journal.pcbi.1005228 (PMC5245781; doi:10.1371/journal.pcbi.1005228)
Supplement: S2 Text — (PDF) [file pcbi.1005228.s002.pdf]

Effect of the latent reservoir on the evolution of HIV at the within- and between-host levels

Supporting Text S2

Hilje M. Doekes[1,2], Christophe Fraser[1,3], Katrina A. Lythgoe[1,4,*]

[1] Department of Infectious Disease Epidemiology, Imperial College London, United Kingdom.

[2] Theoretical Biology, Utrecht University, The Netherlands.

[3] Oxford Big Data Institute, Li Ka Shing Centre for Health Information and Discovery, Nuffield Department of Medicine, University of Oxford, United Kingdom.

[4] Department of Zoology, University of Oxford, United Kingdom.

* katrina.lythgoe@zoo.ox.ac.uk.

Modelling preferential transmission of ancestral virus

It has been argued that upon transmission of HIV, ancestral virus might be preferentially transmitted. We adjusted the within-host and between-host model to incorporate this possibility. We assume that during the within-host evolutionary process the virus acquires certain mutations that lower its transmissibility relative to the other viral strains present in the host, up to a certain maximum number of mutations m. The overall transmissibility of a host is not affected by these mutations. In addition, these mutations are assumed to be neutral at the within-host level, and are not linked to the mutations that increase the replication rate and/or virulence.

Let $x_j^k(\tau)$ and $y_j^k(\tau)$ be the frequency of strain j carrying k of these transmissibility-lowering mutations in the active compartment and the reservoir, respectively. Define the vectors

$$\boldsymbol{x} = (x_1^0, x_1^1, \dots, x_1^m, x_2^0, x_2^1, \dots, x_n^m)\,, \tag{S2.1}$$

$$\boldsymbol{y} = (y_1^0, y_1^1, \dots, y_1^m, y_2^0, y_2^1, \dots, y_n^m)\,. \tag{S2.2}$$

Let $\mu_{\theta+}$ be the rate at which transmissibility-lowering mutations occur during replication, and let these mutations be reverted at rate $\mu_{\theta-}$. The within-host system is then again described by *Eq 2* (main text), however now with

$$Q = \begin{pmatrix} \tilde{q}_{11}J & \cdots & \tilde{q}_{1n}J \\ \vdots & \ddots & \vdots \\ \tilde{q}_{n1}J & \cdots & \tilde{q}_{nn}J \end{pmatrix}, \tag{S2.3}$$

where $\tilde{q}_{ij} = m_{ij}\gamma_j$ is the entry in the original replication-mutation matrix and

$$J = \begin{pmatrix} (1-\mu_{\theta+}) & \mu_{\theta-} & 0 & \cdots & 0 & 0 \\ \mu_{\theta+} & (1-\mu_{\theta+}-\mu_{\theta-}) & \mu_{\theta-} & \cdots & 0 & 0 \\ \vdots & \vdots & \vdots & \ddots & \vdots & \vdots \\ 0 & 0 & 0 & \cdots & \mu_{\theta+} & (1-\mu_{\theta-}) \end{pmatrix} \quad \text{(S2.4)}$$

represents the acquisition of transmissibility-lowering mutations within a strain. The initial infecting strain is assumed to carry none of the transmissibility-lowering mutations, such that for a type-j infection at $\tau = 0$, $x_j^0(0) = y_j^0(0) = 1$ and all other vector entries are zero. Using these initial conditions, the within-host equations can again be numerically integrated to calculate the frequencies of strain i carrying k transmissibility-lowering mutations in the active compartment and the reservoir, $x_{ij}^k(\tau)$ and $y_{ij}^k(\tau)$, for an infection established by strain j at time $\tau = 0$.

Next, we again define the strain specific infectivity profile $\beta_{ij}(\tau)$. The relative contribution of strains to the infectivity now depends both on their frequency and the number of transmissibility-lowering mutations they carry. Let θ_k represent the relative transmissibility of a strain with k transmissibility-lowering mutations compared to a strain without these mutations (i.e. $\theta_0 = 1$). Then, we can define

$$\beta_{ij}(\tau) = \alpha_j(t) \frac{\sum_{k=0}^{m} \theta_k x_{ij}^k(\tau)}{\sum_{i=1}^{n} \sum_{k=0}^{m} \theta_k x_{ij}^k(\tau)}. \quad \text{(S2.5)}$$

Here, the denominator ensures that the total infectivity does not reduce over time. Only the relative transmissibility of strains carrying a certain number of transmissibility-lowering mutations is affected. Relative transmissibility is assumed to decrease linearly with the number of transmissibility-lowering mutations, up to a certain minimum θ_{min}. I.e.

$$\theta_k = \max(\theta_{min}, 1 - k\theta_{step}), \quad \text{(S2.6)}$$

where θ_{step} is the slope of the decrease. The strain specific infectivity profiles as described by *Eq S2.5* were used to directly implement the preferential transmission mechanism in the between-host model described by *Eqs 8-11* (main text).
