# Supplementary material for: Effect of the Latent Reservoir on the Evolution of HIV at the Within- and Between-Host Levels
Source: PLoS Comput Biol. 2017 Jan 19;13(1):e1005228. doi: 10.1371/journal.pcbi.1005228 (PMC5245781; doi:10.1371/journal.pcbi.1005228)
Supplement: S1 Table — (PDF) [file pcbi.1005228.s003.pdf]

**Supplementary Table 1.** Estimates of the relative reservoir size $r_L$.

**DIRECT ESTIMATES**

*Chun et al., Nature, 1997* — $\boldsymbol{P_a}$ = 0.4

| Donor | Active CD4 T cells with integrated HIV DNA (per $10^6$ cells) | Resting CD4 T cells with integrated HIV DNA (per $10^6$ cells) | Estimated $r_L$ |
|---|---|---|---|
| 4 | 387 | 92 | 0.36 |
| 5 | 101 | 96 | 1.43 |
| 6 | 257 | 29 | 0.17 |
| 8 | 172 | 96 | 0.84 |
| 9 | 598 | 62 | 0.16 |
| 10 | 345 | 62 | 0.27 |
| 12 | 80 | 17 | 0.32 |
| ***Median*** | ***257*** | ***62*** | ***0.32*** |

**INDIRECT ESTIMATES**

*Ibáñez et al., AIDS, 1999.*

| Subject | $CD4^+$ T cell count pre-ART (cell/ml) | Integrated HIV-1 DNA copies pre-ART (per $10^6$ PBMCs) | Integrated HIV-1 DNA copies pre-ART (~per ml) | $CD4^+$ T cell count during ART (cell/ml) | Integrated HIV-1 DNA copies during ART (per $10^6$ PBMCs) | Integrated HIV-1 DNA copies during ART (~per ml) | Estimated $r_L$ |
|---|---|---|---|---|---|---|---|
| 1 | 1147 | 113 | 0.130 | 1128 | 87 | 0.098 | 3.1 |
| 2 | 471 | 198 | 0.093 | 748 | 30 | 0.022 | 0.32 |
| 3 | 607 | 73 | 0.044 | 928 | 173 | 0.161 | -* |
| 4 | 660 | 1000 | 0.660 | 1012 | 198 | 0.200 | 0.44 |
| 5 | 616 | 233 | 0.144 | 1016 | 33 | 0.034 | 0.30 |
| 6 | 439 | 280 | 0.123 | 935 | 960 | 0.898 | -* |
| 7 | 300 | 467 | 0.140 | 560 | 133 | 0.074 | 1.14 |
| 8 | 919 | 453 | 0.416 | 1212 | 30 | 0.036 | 0.10 |
| 9 | 435 | 53 | 0.023 | 807 | 87 | 0.070 | -* |
| 10 | 660 | 198 | 0.131 | 940 | 327 | 0.307 | -* |
| ***Median*** | ***611.5*** | ***215.5*** | ***0.130*** | ***937.5*** | ***110*** | ***0.086*** | ***0.38*** |

*Koelsch et al., JID, 2008.*

| Subject | HIV DNA level pre-ART (per μg DNA) | HIV DNA level during ART (per μg DNA) | Estimated $r_L$ |
|---|---|---|---|
| C1 | 100 | 125.9 | -* |
| C2 | 35.5 | 12.6 | 0.55 |
| C3 | 398.1 | 89.1 | 0.29 |
| C4 | 446.7 | 25.1 | 0.06 |
| C5 | 398.1 | 63.1 | 0.19 |
| C6 | 708.0 | 177.8 | 0.34 |
| C7 | 251.2 | 17.8 | 0.08 |
| ***Median*** | ***398.11*** | ***63.10*** | ***0.24*** |

*Chomont et al., Nat Med, 2009.*

| Subject | Integrated HIV DNA pre-ART (per $10^6$ $CD4^+$ T cells) | Integrated HIV DNA during ART (per $10^6$ $CD4^+$ T cells) | Estimated $r_L$ |
|---|---|---|---|
| A | 19952 | 1259 | 0.07 |
| D | 3981 | 562 | 0.16 |
| K | 2511 | 1000 | 0.66 |
| L | 7943 | 1259 | 0.19 |
| ***Median*** | ***5962*** | ***1129*** | ***0.18*** |

*Andreoni et al., AIDS, 2000.*

| Group† | $CD4^+$ count pre-ART (cell/ml) | HIV-DNA copies pre-ART (per $10^6$ CD4+ T cells) | HIV-DNA copies pre-ART (per ml) | $CD4^+$ count during ART (cell/ml) | HIV-DNA copies during ART (per $10^6$ CD4+ T cells) | HIV-DNA copies during ART (per ml) | Estimated $r_L$ |
|---|---|---|---|---|---|---|---|
| 1 | 756 | 288 | 0.218 | 1040 | 17 | 0.018 | 0.09 |
| 2 | 737 | 676 | 0.498 | 920 | 50 | 0.046 | 0.10 |
| 3 | 394 | 2570 | 1.012 | 600 | 161 | 0.096 | 0.11 |
| 4 | 70 | 6761 | 0.473 | 300 | 974 | 0.292 | 1.61 |

*Ngo-Giang-Huong et al., AIDS, 2001.*

| | HIV DNA copies pre-ART (per $10^6$ PBMCs) | HIV DNA copies during ART (per $10^6$ PBMCs) | Estimated $r_L$ |
|---|---|---|---|
| $1^{st}$ Q | 794 | 316 | 0.66 |
| median | 3548 | 1144 | 0.46 |
| $3^{rd}$ Q | 7943 | 2512 | 0.46 |

* $r_L$ could not be calculated because $[HIV_{DNA}]_t > [HIV_{DNA}]_n$; † Groups defined based on CD4 count (high/low) and viral load (high/low), see Andreoni et al.
